# Quantum Teleportation and Dense Coding in Multiple Bosonic Reservoirs

**DOI:** 10.3390/e24081114

**Published:** 2022-08-12

**Authors:** Yu Wang, Ming-Liang Hu

**Affiliations:** 1 School of Electronic Engineering, Xi’an University of Posts and Telecommunications, Xi’an 710121, China; 2 School of Science, Xi’an University of Posts and Telecommunications, Xi’an 710121, China

**Keywords:** quantum teleportation, dense coding, non-Markovianity, 03.65.Ta, 03.65.Yz, 03.67.Hk

## Abstract

The effect of a reservoir on quantum communication depends on its spectral density. The efficiency of quantum teleportation and dense coding is explored when each one of the channel qubits is coupled simultaneously to multiple bosonic reservoirs. It is shown that the non-Markovianity triggered by increasing the reservoir number can induce revivals of quantum advantages of the two protocols after their disappearance. However, the backflow of information to the system that signifies non-Markovianity does not always induce immediate revivals of the quantum advantages. There may be a delayed effect for some initial states, and only as the backflow of information accumulates to a certain extent can the revivals of quantum advantages be triggered.

## 1. Introduction

Quantum communication protocols outperform their classical counterparts in many aspects, e.g., they have high security and channel capacity [1]. Among the various protocols, quantum teleportation is an archetype which uses the prior shared entanglement between the sender Alice and receiver Bob as a physical resource [2]. It enables the disembodied transmission of an unknown state by local operations and classical communication and gives unity fidelity when the shared channel state is maximally entangled [2]. For a general entangled channel state, this protocol shows a quantum advantage when the average fidelity is larger than the classical limiting value 2/3 [3,4]. Another well-known communication protocol is dense coding which also uses entanglement as a resource [5,6,7]. Different from quantum teleportation, there is one qubit being sent from Alice to Bob, and this enables the transmission of two bits of classical information if the channel state is maximally entangled. For certain non-maximally entangled states, this protocol can also show a quantum advantage, that is, it may achieve a dense coding capacity that is not achievable by any classical manner [8].

The quantum advantages of teleportation and dense coding strongly depend on the shared entanglement [9,10,11,12], which is very fragile and might degrade rapidly due to the unavoidable interaction of the system with its environment [13]. Based on this consideration, the effects of different noises on teleportation [14,15,16,17,18] and dense coding [19,20,21,22,23] have been extensively studied. It was shown that their quantum advantages may exist for a relatively long time by choosing specific channel states [24,25], by using the desired features (the non-Markovianity, the correlated actions, etc.) of the noises [26,27,28], or by applying active operations, such as local filtering operations [27] and weak measurements [29,30].

An important research direction concerning environmental effects is the case of the qubits being immersed in reservoirs. In such a scenario, the decay behaviors of entanglement have been extensively studied [31,32,33,34,35,36]. Similarly, the behaviors of discord-like correlations in bosonic reservoirs have also been studied [37,38,39,40]. These works focus on the cases in which the two qubits are coupled to two independent reservoirs or to a common reservoir, and the results show that the non-Markovianity triggered by tuning the spectral density of the reservoir is beneficial for protecting quantum correlations.

In this paper, we consider quantum teleportation and dense coding in the bosonic reservoirs. For a single qubit coupled to *N* reservoirs (i.e., the multiple reservoirs), it has been shown that the non-Markovianity will be triggered when *N* becomes larger than a threshold [41]. Here, we go one step further from the single-qubit case to the two-qubit case. Specifically, we suppose the two qubits serving as the quantum channel are subject to two independent groups of multiple reservoirs. We will focus on their efficiency for implementing the teleportation and dense coding protocols. Different from one’s intuition that the addition of a reservoir introduces additional decoherence, the results show that by increasing the number of involved reservoirs, one can observe revivals of the lost quantum advantages for teleportation and dense coding, although for certain initial states, the revivals do not occur synchronously with the occurrence of the backflow of information from the reservoirs to the system. This shows the complex effects of the multiple reservoirs on the implementation of the quantum protocols and may be useful for protecting the quantum advantages of the teleportation and dense coding protocols.

## 2. Preliminaries

In this section, we recall the quantifiers that will be used to characterize the efficiency of the quantum channel for implementing the tasks of quantum teleportation and dense coding. Both these two tasks show quantum advantages over their classical counterparts if the qubits constituting the quantum channel are entangled in a certain way.

We first consider the teleportation of an unknown state $|\varphi_{\mathrm{in}}\rangle$ from Alice to Bob, which can be implemented by Alice’s local operations on her qubits (i.e., the qubit encoding $|\varphi_{\mathrm{in}}\rangle$ and the channel qubit at her hand) and Bob’s recovery operation on the channel qubit at his hand based on the classical information received from Alice (i.e., Alice’s measurement outcomes) [2]. Suppose Bob’s recovered state is $\rho_{\mathrm{out}}$, then the quality of teleportation could be quantified by the fidelity $F=\langle \varphi_{\mathrm{in}}|\rho_{\mathrm{out}}|\varphi_{\mathrm{in}}\rangle$, which depends on $|\varphi_{\mathrm{in}}\rangle$. To characterize efficiency of the quantum channel for teleportation, one needs to consider the average effect of all possible $|\varphi_{\mathrm{in}}\rangle$, that is, by averaging *F* over all possible $|\varphi_{\mathrm{in}}\rangle$. If $|\varphi_{\mathrm{in}}\rangle$ is the single-qubit state and an arbitrary two-qubit state $\rho_{AB}$ is used as the quantum channel, the average fidelity of teleportation optimized over the local quantum and classical communication operations can be obtained as [3]

(1) $$F_{av}\left(\rho_{AB}\right)=\frac{1}{2}+\frac{1}{6}N\left(\rho_{AB}\right),$$

where $N\left(\rho_{AB}\right)=\mathrm{tr}\sqrt{T^{†}T}$ is the trace norm of the correlation tensor *T* with elements $T_{ij}=\mathrm{tr}\left(\rho_{AB}\sigma_{i}⊗\sigma_{j}\right)$, and $\sigma_{i}$ (i=1,2,3) are the Pauli operators. It has also been shown that the average fidelity equals $\left(F_{\max}d+1\right)/\left(d+1\right)$, with *d* being the dimension of $|\varphi_{\mathrm{in}}\rangle$ and $F_{\max}$ the maximal singlet fraction which is smaller than or equal to 1/d for the separable states, so the best average fidelity via classical channel is 2/3 for d=2 [42]. For certain entangled $\rho_{AB}$, $F_{av}\left(\rho_{AB}\right)$ can exceed this classical limiting value; thus, quantum teleportation outperforms the purely classical protocols and shows quantum advantage [4].

Next, we recall the quantifier for characterizing dense coding. Different from quantum teleportation that is implemented without sending any particle physically, dense coding refers to the protocol with Alice’s channel qubit being sent to Bob after her local unitary transformation. The purpose of this protocol is to show quantum advantage of $\rho_{AB}$ for transmitting classical information, the optimal amount of which is bounded from above by the Holevo quantity [8,43]

(2) $$\chi \left(\rho_{AB}\right)=S\left({\bar{\rho }}_{AB}\right)-S\left(\rho_{AB}\right),$$

where ${\bar{\rho }}_{AB}=\frac{1}{4}\sum_{k=0}^{3}\rho_{AB}^{k}$ is the ensemble state averaged over the signal states $\rho_{AB}^{k}=\left(\sigma_{k}⊗𝟙\right)\rho_{AB}\left(\sigma_{k}⊗𝟙\right)$ ($\sigma_{0}=𝟙$ represents the identity operator), while $S\left({\bar{\rho }}_{AB}\right)=-\mathrm{tr}\left({\bar{\rho }}_{AB}\log_{2}{\bar{\rho }}_{AB}\right)$ is the von Neumann entropy of ${\bar{\rho }}_{AB}$, and likewise for $S(\rho_{AB})$. Because the Holevo quantity is asymptotically achievable [44], one can dub $\chi (\rho_{AB})$ as the dense coding capacity. This protocol shows quantum advantage when $\chi (\rho_{AB})>1$, and one has $\chi (\rho_{AB})=2$ when $\rho_{AB}$ is maximally entangled.

## 3. Solution of the Model

Suppose the qubits *A* and *B*, serving as the quantum channel, are individually submerged in two groups of multiple bosonic reservoirs, then the single “qubit+reservoirs" Hamiltonian ${\hat{H}}_{S}$ (S=A,B) can be written as

(3) $${\hat{H}}_{S}=\frac{1}{2}\omega_{0}\sigma_{z}+\sum_{n=1}^{N_{S}}\left(\sum_{k}\omega_{n,k}b_{n,k}^{†}b_{n,k}+\sigma_{+}B_{n}+\sigma_{-}B_{n}^{†}\right),$$

where $B_{n}=\sum_{k}g_{n,k}b_{n,k}$ with $g_{n,k}$ being the coupling strengths, the index *k* labels the *n*th reservoir’s field mode with frequency $\omega_{n,k}$, and $b_{n,k}$ ($b_{n,k}^{†}$) is the corresponding annihilation (creation) operator. Moreover, $\omega_{0}$ is the transition frequency of the qubit and $\sigma_{\pm }=\left(\sigma_{1}\pm i\sigma_{2}\right)/2$ are the raising and lowering operators. Such a multiple interaction could be realized by placing a two-level atom at the center of $N_{S}$ lossy cavities [33,41,45]. We suppose that there is no initial correlation between the qubit and the reservoirs, and then by tracing out the $N_{S}$ reservoirs, the evolving state of qubit *S* in the basis {|1〉,|0〉} can be obtained as [33,41]

(4) $$\rho_{S}\left(t\right)=\left(\begin{array}{cc} \rho_{S}^{11}\left(0\right){\left|q_{S}\right|}^{2} & \rho_{S}^{10}\left(0\right)q_{S}\;\\ \rho_{S}^{01}\left(0\right)q_{S}^{*} & 1-\rho_{S}^{11}\left(0\right){\left|q_{S}\right|}^{2} \end{array}\right),$$

where $\rho_{S}^{ij}\left(0\right)=\langle i|\rho_{S}\left(0\right)|j\rangle$ with $\rho_{S}\left(0\right)$ being the initial state of qubit *S*, and $q_{S}$ is a time-dependent parameter determined by the spectra of the multiple reservoirs.

For two qubits *A* and *B* coupled independently to two sets of multiple reservoirs, their evolving state $\rho_{AB}\left(t\right)$ at time *t* can be obtained from Equation (4) [31,46]. Here, we consider the case that they are prepared initially in one of the Bell-like states:

(5) |Ψ〉=α|10〉+β|01〉,|Φ〉=α|11〉+β|00〉.

For the initial state |Ψ〉, the nonzero elements of $\rho_{AB}\left(t\right)$ can be obtained as

(6) $$\begin{array}{cc} \; & \rho_{AB}^{22}\left(t\right)={\left|\alpha q_{A}\right|}^{2},\;\rho_{AB}^{23}\left(t\right)={\left[\rho_{AB}^{32}\left(t\right)\right]}^{*}=\alpha \beta^{*}q_{A}q_{B}^{*},\\ \; & \rho_{AB}^{33}\left(t\right)=|\beta q_{B}{|}^{2},\;\rho_{AB}^{44}\left(t\right)=1-|\alpha q_{A}{|}^{2}-{\left|\beta q_{B}\right|}^{2}, \end{array}$$

based on which one can obtain the average fidelity of teleportation and the dense coding capacity as

(7) $$\begin{array}{cc} \; & F_{av}\left(\rho_{AB}\right)=\frac{1}{2}+\frac{2}{3}|\alpha \beta q_{A}q_{B}|+\frac{1}{6}|1-2(|\alpha q_{A}{|}^{2}+|\beta q_{B}{|}^{2})|,\\ \; & \chi \left(\rho_{AB}\right)=1+H_{2}(|\beta q_{B}{|}^{2})-H_{2}(|\alpha q_{A}{|}^{2}+|\beta q_{B}{|}^{2}), \end{array}$$

where $H_{2}\left(x\right)=-x\log_{2}x-\left(1-x\right)\log_{2}\left(1-x\right)$ denotes the binary Shannon entropy function.

Similarly, for the initial state |Φ〉, the nonzero elements of $\rho_{AB}\left(t\right)$ can be obtained as

(8) $$\begin{array}{cc} \; & \rho_{AB}^{11}\left(t\right)={\left|\alpha q_{A}q_{B}\right|}^{2},\;\rho_{AB}^{14}\left(t\right)={\left[\rho_{AB}^{41}\left(t\right)\right]}^{*}=\alpha \beta^{*}q_{A}q_{B},\\ \; & \rho_{AB}^{22}\left(t\right)=|\alpha q_{A}{|}^{2}\left(1-\right|q_{B}{|}^{2}),\;\rho_{AB}^{33}\left(t\right)=|\alpha q_{B}{|}^{2}\left(1-\right|q_{A}{|}^{2}),\\ \; & \rho_{AB}^{44}\left(t\right)=1-{\left|\alpha \right|}^{2}\left(\right|q_{A}{|}^{2}+|q_{B}{|}^{2}-|q_{A}q_{B}{|}^{2}), \end{array}$$

then by denoting $\varepsilon_{\pm }=\rho_{AB}^{11}\left(t\right)\pm \rho_{AB}^{44}\left(t\right)$, the eigenvalues of $\rho_{AB}\left(t\right)$ given in Equation (8) can be obtained as

(9) $$\epsilon_{1}=\rho_{AB}^{22}\left(t\right),\;\epsilon_{2}=\rho_{AB}^{33}\left(t\right),\;\epsilon_{3,4}=\frac{\varepsilon_{+}\pm \sqrt{\varepsilon_{-}^{2}+4|\rho_{AB}^{14}\left(t\right){|}^{2}}}{2},$$

hence, one has

(10) $$\begin{array}{cc} \; & F_{av}\left(\rho_{AB}\right)=\frac{1}{2}+\frac{2}{3}|\alpha \beta q_{A}q_{B}|+\frac{1}{6}\left|1-2\left(\epsilon_{1}+\epsilon_{2}\right)\right|,\\ \; & \chi \left(\rho_{AB}\right)=1+H_{2}(|\alpha q_{B}{|}^{2})+\sum_{n=1}^{4}\epsilon_{n}\log_{2}\epsilon_{n}. \end{array}$$

## 4. Behaviors of Average Fidelity and Dense Coding Capacity

**Figure 1:**
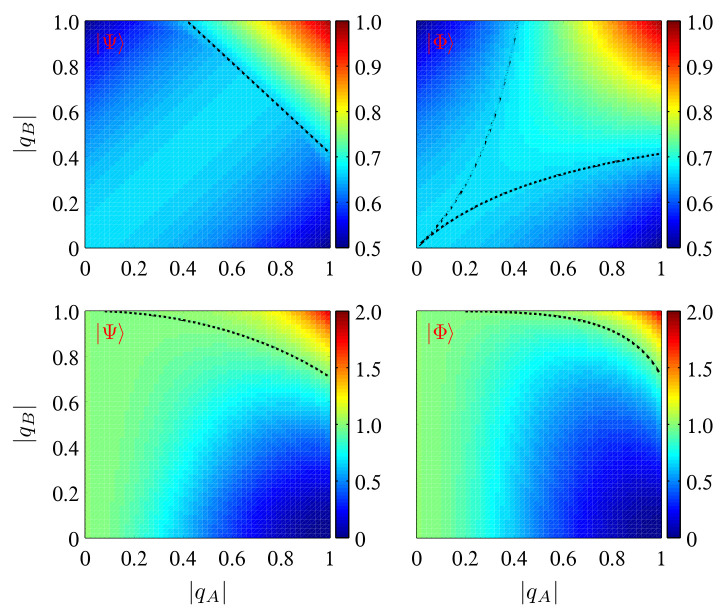
Average fidelity of teleportation $F_{av}$ (the top two panels) and dense coding capacity χ (the bottom two panels) versus $|q_{A}|$ and $|q_{B}|$ for the initial states |Ψ〉 and |Φ〉 with $\alpha =\beta =1/\sqrt{2}$. In the top right corner of the black lines, the teleportation and dense coding protocol show quantum advantages.

For the given initial states, one can see from Equations (7) and (10) that both $F_{av}\left(\rho_{AB}\right)$ and $\chi (\rho_{AB})$ are determined by $|q_{A}|$ and $|q_{B}|$. By choosing $\alpha =\beta =1/\sqrt{2}$, we show in Figure 1 their dependence on $|q_{A}|$ and $|q_{B}|$, from which one can note that $F_{av}\left(\rho_{AB}\right)$ is symmetric with respect to $|q_{A}\left|=\right|q_{B}|$. For the initial state |Ψ〉, $F_{av}\left(\rho_{AB}\right)=2/3-{\left|q_{A}\right|}^{2}/6$ when $|q_{B}|=0$, which decreases monotonically from 2/3 to 1/2 with the increase of $|q_{A}|$. When $|q_{B}{|}^{2}\in \left(0,1/2\right)$, $F_{av}\left(\rho_{AB}\right)$ first increases to its peak value of 2/3 and then decreases to a minimum, after which it increases gradually to $1/3+\left(\right|q_{B}{\left|+1\right)}^{2}/6$ at $|q_{A}|=1$. Finally, for $|q_{B}{|}^{2}>1/2$, $F_{av}\left(\rho_{AB}\right)$ always increases with an increase of $|q_{A}|$. When considering |Φ〉, one still has $F_{av}\left(\rho_{AB}\right)=2/3-{\left|q_{A}\right|}^{2}/6$ when $|q_{B}|=0$. However, when $|q_{B}|\in \left(0,1/2\right)$, $F_{av}\left(\rho_{AB}\right)$ first increases to a peak value larger than 2/3 and then decreases to a minimum, and when $|q_{B}|>1/2$, $F_{av}\left(\rho_{AB}\right)$ always increases monotonically with the increase of $|q_{A}|$. Moreover, as is shown in the top two panels of Figure 1, $F_{av}\left(\rho_{AB}\right)$ for the initial state |Φ〉 exceeds the classical limiting value of 2/3 in an extended region of $\left(|q_{A}|,|q_{B}|\right)$ compared to that for |Ψ〉.

For the dense coding capacity $\chi (\rho_{AB})$, as can be seen from the bottom two panels of Figure 1, it is asymmetric with respect to $|q_{A}\left|=\right|q_{B}|$. We analyze its behaviors for the initial state |Ψ〉 (its behaviors are structurally the same for |Φ〉). For $|q_{B}|=0$, $\chi \left(\rho_{AB}\right)=1-H_{2}\left(\right|q_{A}{|}^{2}/2)$, which decreases from 1 to 0 when $|q_{A}|$ increases from 0 to 1. For any fixed $|q_{B}|\in \left(0,1\right)$, however, $\chi (\rho_{AB})$ first decreases to a minimum and then turns to be increased gradually, and when $|q_{B}{|}^{2}>1/2$, one has $\chi (\rho_{AB})>1$ in the region of $|q_{A}{|}^{2}>2(1-|q_{B}{|}^{2})$. Finally, for $|q_{B}|=1$, one has $\chi \left(\rho_{AB}\right)=2-H_{2}\left[(1+\right|q_{A}{|}^{2})/2]$, which increases gradually from 1 to 2 when $|q_{A}|$ increases from 0 to 1. On the other hand, for $|q_{A}|=0$, one has $\chi (\rho_{AB})\equiv 1$, and for any fixed $|q_{A}|>0$, it increases monotonically with the increase of $|q_{B}|$ and shows quantum advantage when $|q_{B}{|}^{2}$ becomes larger than $1-|q_{A}{|}^{2}/2$. Moreover, different from that of $F_{av}\left(\rho_{AB}\right)$, the initial state |Ψ〉 yields a wider region of $\left(|q_{A}|,|q_{B}|\right)$ than that of |Ψ〉 in which $\chi (\rho_{AB})>1$.

In the following, we give some explicit examples of the multiple bosonic reservoirs with different spectra to demonstrate the time dependence of $F_{av}\left(\rho_{AB}\right)$ and $\chi (\rho_{AB})$, as well as their connections with the flow of information between the system and the multiple reservoirs. Here, the spectral density of the multiple reservoirs can be written as $J\left(\omega \right)=\sum_{n=1}^{N_{S}}J_{n}\left(\omega \right)$, with $J_{n}\left(\omega \right)$ being the spectral density of the *n*th reservoir. For simplicity, we concentrate on the case that all the reservoirs are the same, i.e., $J_{n}\left(\omega \right)\equiv J\left(\omega \right)$, ∀n.

### 4.1. Lorentzian Spectrum

First, we consider the multiple bosonic reservoirs with the Lorentzian spectrum [47]

(11) $$J\left(\omega \right)=\frac{1}{2\pi }\frac{\gamma \lambda^{2}}{{\left(\omega -\omega_{0}\right)}^{2}+\lambda^{2}},$$

where λ and γ represent, respectively, the spectral width of the reservoir and the decay rate of the qubit. They are related to the reservoir’s correlation time $\tau_{B}$ and relaxation time $\tau_{R}$ by $\tau_{B}≃\lambda^{-1}$ and $\tau_{R}≃\gamma^{-1}$ [47].

For such a spectral density, the decoherence factor $q_{S}\left(t\right)$ can be obtained as [31]

(12) $$q_{S}\left(t\right)=e^{-\frac{1}{2}\lambda t}\left(\cosh\frac{d_{S}t}{2}+\frac{\lambda }{d_{S}}\sinh\frac{d_{S}t}{2}\right),$$

where $d_{S}={\left(\lambda^{2}-2N_{S}\gamma \lambda \right)}^{1/2}$. Then, one can see that when $\lambda >2N_{S}\gamma$, $q_{S}\left(t\right)$ decays exponentially with time and the evolution is Markovian. However, when $\lambda <2N_{S}\gamma$, $d_{S}$ becomes an imaginary number and $q_{S}\left(t\right)$ oscillates with time; hence, the evolution will be non-Markovian. For given λ and γ, there exists a threshold $N_{S,\mathrm{cr}}=\left\lfloor \lambda /2\gamma \right\rfloor +1$ (⌊x⌋ is the nearest integer not larger than *x*). The non-Markovianity occurs when $N_{S}⩾N_{S,\mathrm{cr}}$.

**Figure 2:**
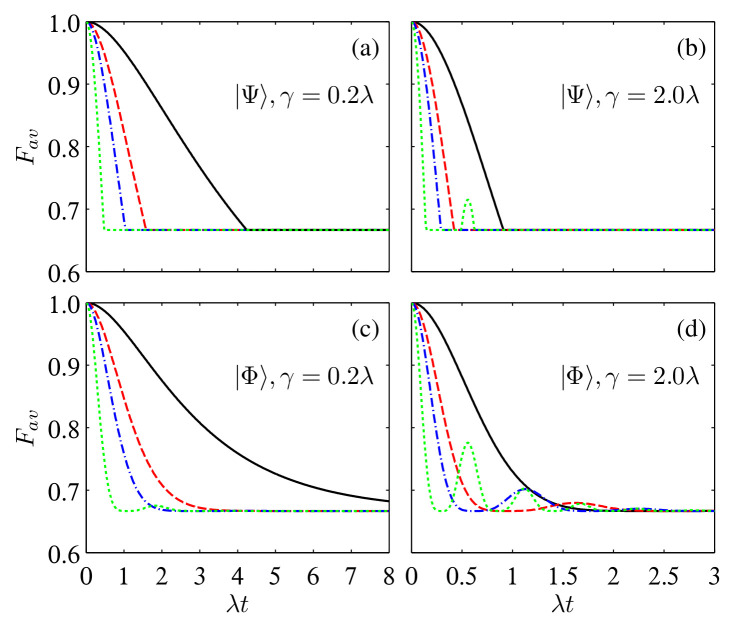
Time evolution of $F_{av}$ in the multiple Lorentzian reservoirs for the initial states |Ψ〉 (**a**,**b**) and |Φ〉 (**c**,**d**) with $\alpha =\beta =1/\sqrt{2}$ and different γ. The solid black, dashed red, dash-dotted blue, and dotted green lines correspond to $N_{A,B}=1$, 4, 8, and 32, respectively.

**Figure 3:**
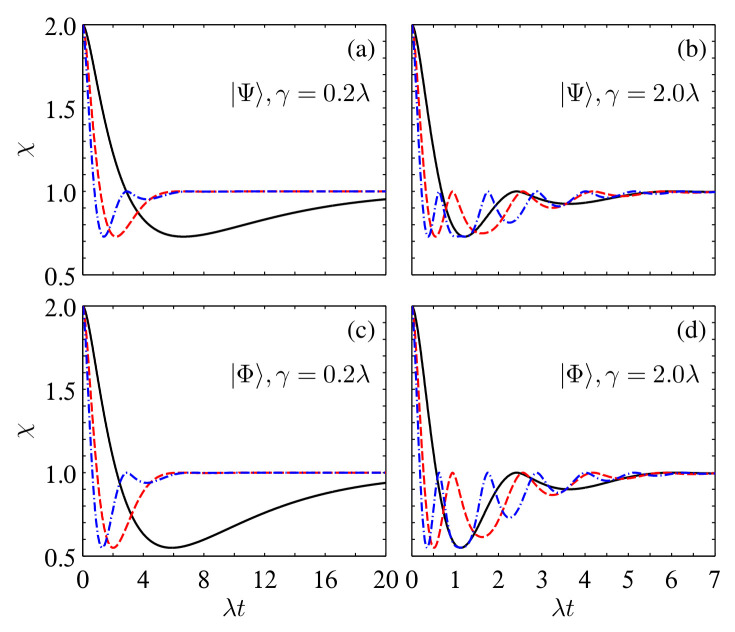
Time evolution of χ in the multiple Lorentzian reservoirs for the initial states |Ψ〉 (**a**,**b**) and |Φ〉 (**c**,**d**) with $\alpha =\beta =1/\sqrt{2}$ and different γ. The solid black, dashed red, and dash-dotted blue lines correspond to $N_{A,B}=1$, 4, and 8, respectively.

By substituting $q_{S}\left(t\right)$ into Equations (7) and (10), one can obtain the time dependence of both $F_{av}\left(\rho_{AB}\right)$ and $\chi (\rho_{AB})$. By choosing $N_{A}=N_{B}$, we display their behaviors in Figure 2 and Figure 3 for the initial states |Ψ〉 and |Φ〉 with $\alpha =\beta =1/\sqrt{2}$ and different γ. For $F_{av}\left(\rho_{AB}\right)$ with the initial state |Ψ〉, it decreases monotonically from 1 to the classical limiting value of 2/3 when γ is weak, and the chosen number of reservoirs does not help to enhance the average fidelity, although the non-Markovian effect has already been triggered when $N_{S}⩾N_{S,\mathrm{cr}}=3$ (a further numerical calculation shows that when $N_{S}>208$, there exists a revival phenomenon in the time evolution process). When γ is strong (e.g., γ=2.0λ for which $N_{S,\mathrm{cr}}=1$), $F_{av}\left(\rho_{AB}\right)$ decays more rapidly than that with weak γ at the initial time, but there is revival phenomenon for the relative small $N_{S}$. For $F_{av}\left(\rho_{AB}\right)$ with the initial state |Φ〉, as is shown in the bottom two panels of Figure 2, the non-Markovianity triggered by increasing the number $N_{S}$ of reservoirs induces revival of $F_{av}\left(\rho_{AB}\right)$ only when $N_{S}$ is large enough, and the amplitude of the revival increases with the increase of $N_{S}$. One can also note that $F_{av}\left(\rho_{AB}\right)$ for the initial state |Φ〉 decays slower than that for |Ψ〉, that is, the former is more efficient for teleporting the one-qubit state than the latter.

When considering $\chi (\rho_{AB})$, its dynamical behaviors with different system parameters are shown in Figure 3. For the Markovian case, $\chi (\rho_{AB})$ first decays from 2 to a minimum value and then increases asymptotically to the classical limiting value of 1. However, for the non-Markovian case, either being triggered by increasing the coupling strength γ or by increasing the number of reservoirs, $\chi (\rho_{AB})$ shows damped oscillations with evolving time *t* and also approaches 1 in the infinite-time limit. In particular, $\chi (\rho_{AB})$ cannot exceed 1 after the first disappearance of $\chi (\rho_{AB})>1$ at a critical time $t_{c}$ for the chosen γ and $N_{S}$ in Figure 3. Looking at Figure 3, one can also observe that $t_{c}$ decreases with an increase in $\gamma N_{S}$. Moreover, we would like to mention that for $\gamma N_{S}>120$ for the initial state |Ψ〉 and $\gamma N_{S}>252$ for |Φ〉, one can still observe a reappearance of $\chi (\rho_{AB})>1$ after its first disappearance in the short-time region (we have not shown the plots here for the conciseness of this paper). This can be understood from Equation (12), as for very large $\gamma N_{S}$, one has $d_{S}≃i{\left(2N_{S}\gamma \lambda \right)}^{1/2}$ and $q_{S}\left(t\right)≃e^{-\lambda t/2}\cos(|d_{S}\left|t/2\right)$; hence, it is possible to observe a revival of $\chi (\rho_{AB})>1$ at the neighborhood of $t_{0}=2\pi /\left|d_{S}\right|$. For example, for the initial state |Ψ〉 with $\gamma N_{S}=200$, one has $t_{0}/\pi =0.1$ (in units of λ) and the numerical calculation shows that the revival of $\chi (\rho_{AB})>1$ occurs in the region of $t_{0}/\pi \in \left(0.0907,0.1097\right)$.

### 4.2. Sub-Ohmic, Ohmic, and Super-Ohmic Spectra

In this subsection, we consider the multiple bosonic reservoirs with the following spectra [48]

(13) $$J\left(\omega \right)=\eta \omega^{s}\omega_{c}^{1-s}e^{-\omega /\omega_{c}}$$

where η and $\omega_{c}$ are the system–reservoir coupling strength and the cutoff frequency, which are related to $\tau_{B}$ and $\tau_{R}$ by $\tau_{B}≃\omega_{c}^{-1}$ and $\tau_{R}≃\eta^{-1}$. The reservoir is said to be sub-Ohmic when s∈(0,1), Ohmic when s=1, and super-Ohmic when s>1.

For these kinds of J(ω), the decoherence factor $q_{S}\left(t\right)$ is determined by [49]

(14) $${\dot{q}}_{S}\left(t\right)+i\omega_{0}q_{S}\left(t\right)+\int_{0}^{t}q_{S}\left(\tau \right)f\left(t-\tau \right)d\tau =0,$$

and by denoting Γ(·) for the usual gamma function, the kernel function f(t−τ) can be integrated as

(15) $$f\left(t-\tau \right)=\int_{-\infty }^{\infty }d\omega J\left(\omega \right)e^{-i\omega (t-\tau )}=\frac{\Gamma \left(s+1\right)N_{S}\eta \omega_{c}^{2}}{{\left[1+i\omega_{c}\left(t-\tau \right)\right]}^{s+1}},$$

then Equation (14) can be rewritten as

(16) $$q_{S}\left(t\right)=1-\int_{0}^{t}\left[i\omega_{0}+\int_{\tau }^{t}f\left(t_{1}-\tau \right)dt_{1}\right]q_{S}\left(\tau \right)d\tau ,$$

where $\int_{\tau }^{t}f\left(t_{1}-\tau \right)dt_{1}$ can be integrated as

(17) $$\int_{\tau }^{t}f\left(t_{1}-\tau \right)dt_{1}=i\Gamma \left(s\right)N_{S}\eta \omega_{c}\left\{\frac{1}{{\left[1+i\omega_{c}\left(t-\tau \right)\right]}^{s}}-1\right\},$$

by the substitution of which into Equation (16), one can obtain a simplified integro-differential equation. This equation can be solved numerically [50].

**Figure 4:**
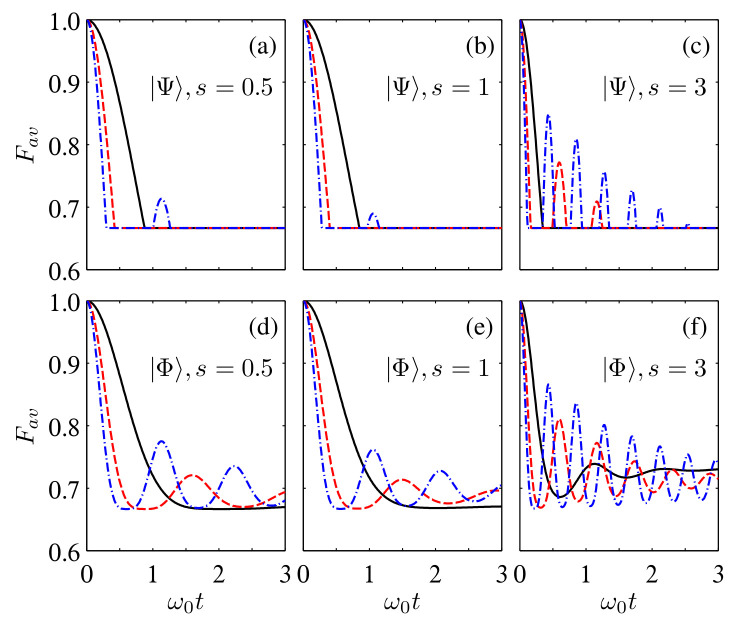
Time evolution of $F_{av}$ in the multiple sub-Ohmic, Ohmic, and super-Ohmic reservoirs for the initial states |Ψ〉 (**a**–**c**) and |Φ〉 (**d**–**f**) with $\alpha =\beta =1/\sqrt{2}$, η=1, and $\omega_{c}=\omega_{0}$. The solid black, dashed red, and dash-dotted blue lines correspond to $N_{A,B}=1$, 4, and 8, respectively.

By fixing $\omega_{c}=\omega_{0}$ and taking s=0.5, 1, and 3 as examples of the sub-Ohmic, Ohmic, and super-Ohmic spectra, we show in Figure 4 time dependence of $F_{av}\left(\rho_{AB}\right)$ for the two initial states |Ψ〉 and |Φ〉. It can be found that for all the considered cases, increasing the number of reservoirs can induce revivals of $F_{av}\left(\rho_{AB}\right)>2/3$. The amplitudes of these damped oscillations could be increased by increasing $N_{S}$, although they approach the classical limiting value of 2/3 in the infinite-time limit. This shows that the non-Markovianity triggered by increasing the number of reservoirs is beneficial for enhancing $F_{av}\left(\rho_{AB}\right)$. Moreover, by comparing the lines in Figure 4 with different *s*, one can note that the times of revivals of $F_{av}\left(\rho_{AB}\right)>2/3$ as well as the amplitudes of the damped oscillations are different for the multiple reservoirs with different spectra. Specifically, while the super-Ohmic (sub-Ohmic) spectrum yields the maximum (minimum) revivals of $F_{av}\left(\rho_{AB}\right)$, the Ohmic spectrum yields the intermediate revivals. Hence, by tuning the value of *s*, one can efficiently tune the decay rate of the average fidelity of quantum teleportation.

**Figure 5:**
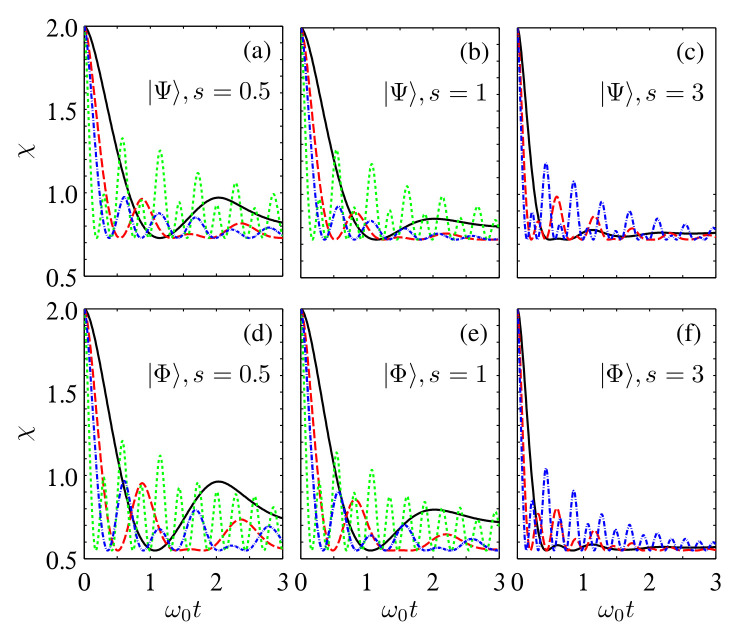
Time evolution of χ in the multiple sub-Ohmic, Ohmic, and super-Ohmic reservoirs for the initial states |Ψ〉 (**a**–**c**) and |Φ〉 (**d**–**f**) with $\alpha =\beta =1/\sqrt{2}$, η=1, and $\omega_{c}=\omega_{0}$. The solid black, dashed red, dash-dotted blue, and dotted green lines correspond to $N_{A,B}=1$, 4, 8, and 32, respectively. To better visualize the plots, we have not shown the dotted green lines for s=3 due to their high frequencies.

Next, we see the dense coding capacity $\chi (\rho_{AB})$, and the corresponding plots are shown in Figure 5. One can see that $\chi (\rho_{AB})$ also shows damped oscillations with evolving time *t*. However, for small $N_{S}$, the dense coding protocol loses its quantum advantage after a short time. By increasing the number of reservoirs acting on each channel qubits, the amplitudes of damped oscillations of $\chi (\rho_{AB})$ are increased. In particular, there are revivals of $\chi (\rho_{AB})>1$ during the time evolution process if $N_{S}$ is larger than a threshold. Moreover, from the point of view of suppressing the decay of $\chi (\rho_{AB})$, the super-Ohmic reservoir outperforms the sub-Ohmic reservoir, and the sub-Ohmic reservoir outperforms the Ohmic reservoir.

We have also performed calculations for other values of $\omega_{c}$. The results show that there is also non-Markovianity being triggered, and both $F_{av}\left(\rho_{AB}\right)$ and $\chi (\rho_{AB})$ show similar dynamical behaviors to those with $\omega_{c}=\omega_{0}$. The difference is that their frequencies of damped oscillations will be increased with an increase of $\omega_{c}$, and they will approach their asymptotic values faster. Thus, for the conciseness of this paper, we do not present the corresponding plots here.

**Figure 6:**
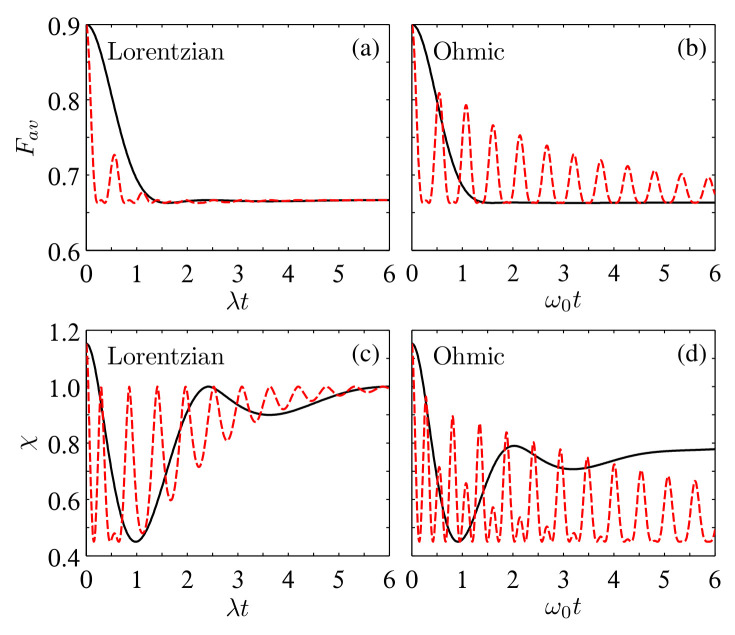
Time evolution of $F_{av}$ (**a**,**b**) and χ (**c**,**d**) in the multiple Lorentzian and Ohmic reservoirs for the initial state $\rho_{W}$ with r=0.8, γ=2.0λ, η=1, and $\omega_{c}=\omega_{0}$. The solid black and dashed red lines correspond to $N_{A,B}=1$ and 32, respectively.

Although we considered in the above two subsections the initial Bell states, the results can be generalized immediately to other initial states. As an example, we show in Figure 6 the time dependence of $F_{av}\left(\rho_{AB}\right)$ and $\chi (\rho_{AB})$ for the initial Werner state $\rho_{W}=r|\Phi^{+}\rangle \langle \Phi^{+}|+\left(1-r\right)𝟙/4$, where $|\Phi^{+}\rangle =(|11\rangle +|00\rangle )/\sqrt{2}$ and r∈[0,1]. One can see that the non-Markovian effect triggered by increasing the reservoir number also induces revivals of the quantum advantages of teleportation. As for dense coding, though there is no revival of the quantum advantage for the chosen system parameters, $\chi (\rho_{AB})$ can also be enhanced noticeably in a wide time region.

From the above discussions, one can see that the dynamical behaviors of $F_{av}\left(\rho_{AB}\right)$ and $\chi (\rho_{AB})$ are intimately related to the non-Markovianity of the multiple bosonic reservoirs. To analyze in detail their relations, we consider the trace distance for two time-evolving states $\rho_{1}\left(t\right)$ and $\rho_{2}\left(t\right)$:

(18) $$D\left[\rho_{1}\left(t\right),\rho_{2}\left(t\right)\right]=\frac{1}{2}\mathrm{tr}\left|\rho_{1}\left(t\right)-\rho_{2}\left(t\right)\right|,$$

the increase in which, with the evolving time, indicates a backflow of information from the reservoirs to the system and the existence of non-Markovianity [51]. For the single-qubit case, the optimal initial states are $|\psi_{1,2}\rangle =(|0\rangle \pm |1\rangle )/\sqrt{2}$; hence, one has $D\left[\rho_{1}\left(t\right),\rho_{2}\left(t\right)\right]=\left|q_{S}\left(t\right)\right|$ [41].

**Figure 7:**
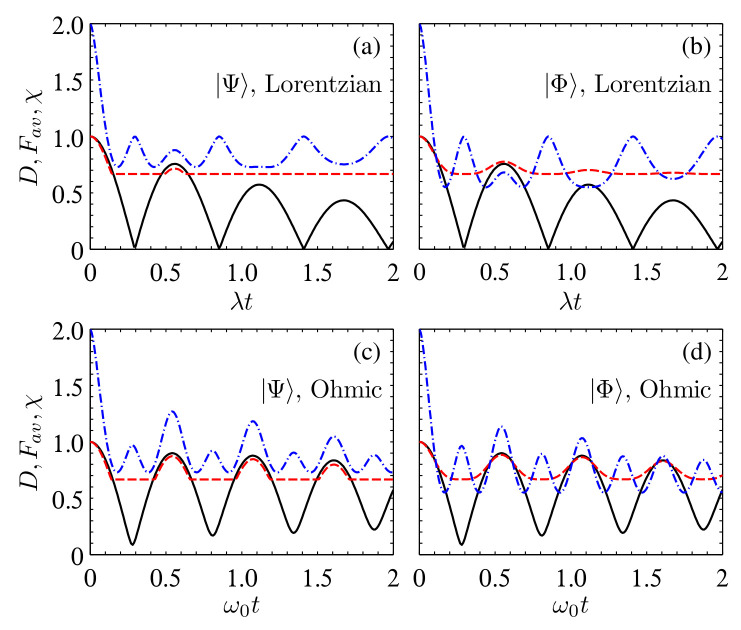
Comparison of the time evolution of *D* (solid black) with $F_{av}$ (dashed red) and χ (dash-dotted blue) in the multiple Lorentzian and Ohmic reservoirs for the initial states |Ψ〉 (**a**,**c**) and |Φ〉 (**b**,**d**) with $\alpha =\beta =1/\sqrt{2}$, γ=2.0λ, η=1, $\omega_{c}=\omega_{0}$, and $N_{A,B}=32$.

In Figure 7, we present a comparison of *D* with $F_{av}\left(\rho_{AB}\right)$ and $\chi (\rho_{AB})$ in the multiple reservoirs with Lorentzian and Ohmic spectra. For the Lorentzian spectrum, *D* suffers instantaneous disappearance at $t_{z,n}=2[n\pi -\arctan(|d_{S}\left|/\lambda )]/\right|d_{S}|$ and reaches to its peak value at $t_{p,n}=2\left(n-1\right)\pi /\left|d_{S}\right|$ (n∈N), while for the Ohmic spectrum, $t_{z,n}$ and $t_{p,n}$ can be obtained numerically. In the time region $t\in (t_{z,n},t_{p,n+1})$, there is a backflow of information from the reservoirs to the system. For the initial state |Ψ〉, the backflow of information does not always induce revivals of $F_{av}\left(\rho_{AB}\right)>2/3$ and $\chi (\rho_{AB})>1$, and there is a delayed effect for the backflow of information on $F_{av}\left(\rho_{AB}\right)$ and $\chi (\rho_{AB})$, that is, the increase of *D* induces revivals of $F_{av}\left(\rho_{AB}\right)>2/3$ and $\chi (\rho_{AB})>1$ only after it increases for a period of time. However, the quantum teleportation and dense coding protocols lose their quantum advantages gradually from the very beginning of the decrease of *D*. For the initial state |Φ〉, the variation of *D* has a similar effect on $\chi (\rho_{AB})$, while it varies synchronously with the variation of $F_{av}\left(\rho_{AB}\right)$.

For the Lorentzian reservoirs, the peak values of $F_{av}\left(\rho_{AB}\right)$ and $\chi (\rho_{AB})$ at $t=t_{p,n}$ can be written explicitly by substituting $q_{S}=e^{\left(1-n\right)\lambda \pi /|d_{S}|}$ into Equations (7) and (10), e.g., $F_{av}\left(\rho_{AB}\right)=\left(1+2\right|q_{S}\left|\right)/3$ for |Ψ〉 and $F_{av}\left(\rho_{AB}\right)=\left(2+\right|q_{S}{|}^{4})/3$ for |Φ〉. Because $D=|q_{S}\left(t\right)|$, this also explains why the increase of *D* does not always induce revivals of $F_{av}\left(\rho_{AB}\right)>2/3$ for |Ψ〉 and why *D* varies synchronously with $F_{av}\left(\rho_{AB}\right)$ for |Φ〉.

In the above discussions, we considered the identical reservoirs for which the resultant effects are equivalent to that obtained by scaling $J_{n}\left(\omega \right)\equiv J\left(\omega \right)$ by $N_{S}$ for a single reservoir. For the general nonidentical reservoirs, i.e., $J_{n}\left(\omega \right)\neq J_{m}\left(\omega \right)$ for n≠m, the expression of $q_{S}\left(t\right)$ will be complicated. For example, for the spectral density $J_{n}\left(\omega \right)=\eta_{n}\omega^{s_{n}}\omega_{c}^{1-s_{n}}e^{-\omega /\omega_{c}}$, after a derivation similar to Equations (15)–(17), one can see that adding multiple reservoirs will not be equivalent to scale $J_{n}\left(\omega \right)$ by $N_{S}$ for a single reservoir, and it is also not equivalent to that obtained by summing the coupling strength of the qubit to each reservoir. However, a further numerical calculation shows that even for this case, the non-Markovianity triggered by adding multiple reservoirs is also beneficial for postponing the decay of the teleportation fidelity and density coding capacity.

## 5. Summary

To summarize, we have investigated the efficiency of quantum teleportation and dense coding for two channel qubits coupled to their respective groups of multiple bosonic reservoirs. For those initial Bell-like states, we showed that both the average fidelity of teleportation and the dense coding capacity are determined by the decoherence factors $q_{S}$ (S=A,B) and we obtained the valid regions of $\left(|q_{A}|,|q_{B}|\right)$ in which the two protocols show quantum advantages. As explicit examples, we further considered the cases that the reservoirs have Lorentzian, sub-Ohmic, Ohmic, and super-Ohmic spectra. It is found that the non-Markovianity, due to the increasing number of reservoirs acting on each channel qubit, can induce revivals of the quantum advantages after their first disappearance, but the explicit processes are different for the teleportation and dense coding protocols. To be explicit, the variation of the trace distance between two optimal initial states (the contractiveness of which signifies the non-Markovian dynamics) does not always vary synchronously with $F_{av}\left(\rho_{AB}\right)$ and $\chi (\rho_{AB})$. There exist circumstances under which there is a delayed effect for the backflow of information on inducing the revivals of the quantum advantages of teleportation and dense coding. These results might provide a way for protecting the quantum advantages of the communication and computation protocols relying on non-Markovian evolutions, e.g., one could use the reservoir engineering technique to experimentally adjust the frequency distribution of a reservoir to the desired regimes [52,53,54,55]. Of course, we considered only the bosonic reservoirs with a single excitation. For the reservoirs whose spectral densities have low-frequency components, [J(ω) is large for $\omega \ll \omega_{0}$], such that one needs to go beyond the single excited subspace, their effects on the considered communication protocols may be different, and a further study on the details is still needed.

## Data Availability

Not applicable.
